# A Thermometrical Table on the Scales of Fahrenheit, Centigrade, and Reaumur

**Published:** 1845-04

**Authors:** 


### A Thermometrical Table on the Scales of Fahrenheit, Centigrade, and Reaumur, &c. By *Alfred S. Taylor,* Lecturer on Chemistry, &c. London, 1845. T. and R. Willats.

We have received this exceedingly instructive " Thermometric Table," by Mr. Alfred Taylor, of Guy's Hospital. It contains a vast deal of information in little space. By its aid, not only can we read off at a glance the corresponding degrees of the three thermometers at present in use—Fahrenheit's, Reaumur's, and the Centrigrade—but we also find out a host of interesting particulars connected with Climatology, Physical Geography, Chemistry, and Physiology. By having it at his side, the student will save himself no little time and trouble in the way of reference to different books.

Would that Mr. Taylor would give us, at his leisure, a similar table of weights and measures, British and Foreign ; so that the reader of French and German works could at once reduce the one into the other.

